# KaScape: a sequencing-based method for global characterization of protein‒DNA binding affinity

**DOI:** 10.1038/s41598-023-43426-x

**Published:** 2023-10-03

**Authors:** Hong Chen, Yongping Xu, Jianshi Jin, Xiao-dong Su

**Affiliations:** 1 State Key Laboratory of Protein and Plant Gene Research, School of Life Sciences, and Biomedical Pioneering Innovation Center (BIOPIC), Peking University, Beijing, 100871 China; 2 State Key Laboratory of Integrated Management of Pest Insects and Rodents, Institute of Zoology, Chinese Academy of Sciences, 1 Beichen West Road, Chaoyang District, Beijing, 100101 People’s Republic of China

**Keywords:** Biochemistry, Biological techniques, Biophysics, Biotechnology, Computational biology and bioinformatics

## Abstract

It is difficult to exhaustively screen all possible DNA binding sequences for a given transcription factor (TF). Here, we developed the KaScape method, in which TFs bind to all possible DNA sequences in the same DNA pool where DNA sequences are prepared by randomized oligo synthesis and the random length can be adjusted to a length such as 4, 5, 6, or 7. After separating bound from unbound double-stranded DNAs (dsDNAs), their sequences are determined by next-generation sequencing. To demonstrate the relative binding affinities of all possible DNA sequences determined by KaScape, we developed three-dimensional KaScape viewing software based on a K-mer graph. We applied KaScape to 12 plant TF family *At*WRKY proteins and found that all *At*WRKY proteins bound to the core sequence GAC with similar profiles. KaScape can detect not only binding sequences consistent with the consensus W-box “TTGAC(C/T)” but also other sequences with weak affinity. KaScape provides a high-throughput, easy-to-operate, sensitive, and exhaustive method for quantitatively characterizing the relative binding strength of a TF with all possible binding sequences, allowing us to comprehensively characterize the specificity and affinity landscape of transcription factors, particularly for moderate- and low-affinity binding sites.

## Introduction

The interaction between transcription factors (TFs) and their specific transcription factor-binding sites (TFBSs)^1^ is critical for TFs to regulate gene expression^2^. The current consensus is that TFs must search along the double-stranded DNA (dsDNA) before they bind to their TFBSs and that there are low- or moderate-affinity binding sites, in addition to specific high-affinity TFBSs. In fact, some moderate- or low-affinity TFBSs may also be necessary for gene regulation^3^. Therefore, a comprehensive understanding of the interaction between TFs and DNA is essential and requires high-throughput (HTP) analytical methods.

The conventional methods EMSA (electrophoretic mobility shift assay) and ITC (isothermal titration calorimetry) have been used for several decades to determine the affinity between TF and TFBS, but it is difficult to study many TFBSs of a specific TF in a high-throughput manner. In recent two decades, high-throughput methods, including both experimental and computational technologies, such as protein binding microarrays (PBMs)^4,5^ and mechanically induced trapping of molecular interactions (MITOMI)^6–8^, have been developed to identify many TFBSs of a specific TF^9–11^. In PBMs, the precise relationship between measured fluorescence intensities and binding energies is unclear, and the number of different sequence probes is limited^8^. In MITOMI, the throughput is limited to a few hundred sequences^8^. Recently, throughput has been improved by techniques such as Binding Energy Topography by sequencing (BET-seq). BET-seq combines MITOMI with high-throughput DNA sequencing. The method focuses on the influence of the flanking sequence around the consensus sequence and is limited to investigating the consensus sequence^8^. These techniques require specific hardware, which prevents their wide application. With the development of HTP, highly accurate, scalable next-generation sequencing (NGS) technologies, NGS has been revolutionizing the study of TFBSs, allowing researchers to investigate the binding events of TFs on a genome-wide scale^9,11,12^. NGS-based methods such as chromatin immunoprecipitation followed by sequencing (ChIP-seq)^13^ and the systematic evolution of ligands by exponential enrichment (SELEX)^14^ have been developed and widely used to identify and map TFBSs across genomes. ChIP-seq is an in vivo method and may produce non-negligible false-positive results; this occurs because the cross-linking step performed in ChIP-seq may cause proteins to become covalently trapped on nonspecific chromosomal DNA, and the antibody used in ChIP-seq may bind nonspecifically to an untargeted TF^15^. The in vitro HTP SELEX technique provides data with less noise. However, SELEX can typically only identify enriched high-affinity TFBSs since it removes low-affinity TFBSs during measurement cycles^16^. Spec-seq is another in vitro method based on sequencing for determining the specificity of protein‒DNA binding^17–19^. Although it can provide binding affinities over a wide range, it can not run millions of sequences in parallel to detect motifs like PBM or SELEX-seq^18^. Spec-seq uses EMSA to separate bound and unbound DNA. However, TF-DNA complexes are not in chemical equilibrium^8^. Instead, complexes with fast dissociation rates may be underrepresented, leading to the underestimation of weak affinity interactions^8^. The design of the Spec-seq experiment is based on the known consensus. Almost all of the methods mentioned above use the position weight matrix (PWM) model to characterize the binding specificity of binding sites, which assumes mononucleotide independence^20,21^.

To overcome the limitations of existing methods, address the need for technologies capable of high-throughput exhaustive thermodynamic measurements, identify the TFBSs of a specific TF including all high- and low-affinity TFBSs simultaneously and further provide a high-quality and exhaustive measurement for understanding the binding mechanism between TFs and DNA, we have developed a new method, KaScape (Ka represents binding affinity and Scape represents landscape), that determines the relative binding affinities of all possible DNA sequences to TFs of interest (for example, *At*WRKY family DNA binding domains) based on NGS. It can measure relative binding energies directly from the experiment, independent of the mononucleotide independence assumption required by PWM. In KaScape, a library containing all possible combinations of 4, 5, 6, or 7 randomized bases of dsDNA sequences, whose composition is determined by NGS, is first mixed with each of the His-tagged *At*WRKY family TFs; second, wash the mixture three times and the bound TF-DNA complexes are quickly separated from the mixture by magnetic His-tag purification beads; third, the DNA sequences in the separated TF-DNA complex pool are determined by NGS; finally, the relative binding affinities of all possible dsDNA sequences are calculated based on both the proportion of each DNA sequence in the separated TF-DNA complex pool and the original DNA library. Furthermore, to visualize and analyze the relative binding affinities of all possible DNA sequences determined by KaScape, we developed a program suite called KGViewer using a K-mer graph in three dimensions.

## Materials and methods

### Randomized dsDNA preparation

We prepared a randomized dsDNA pool by extending another DNA strand onto random single-strand DNAs (ssDNAs) with randomized combinations of 4, 5, 6, or 7 bases in the middle and fixed sequences at both flanking ends using a primer (see Table S1 Complementary ssDNA). The ssDNAs were synthesized by Integrated DNA Technologies, USA (see Table S1 Random ssDNA, where n represents the random base length). The ssDNAs were mixed with the primer (synthesized by Sangon, China) and EasyTaq PCR SuperMix (reagent concentrations are listed in Table S2). The mixture was then incubated using a thermocycler for polymerase chain reaction (PCR) with the program shown in Table S3. The dsDNAs were purified by gel filtration using Superdex75 (GE Healthcare, USA). The purified dsDNAs are referred to as the random dsDNA pool. We note that the length of the dsDNAs is approximately 30 base pairs.

### Protein preparation

The N- or C-terminal DNA-binding domains (DBD) of the *Arabidopsis* WRKY family proteins (*At*WRKY1, *At*WRKY2, *At*WRKY3, *At*WRKY4, *At*WRKY32, and *At*WRKY33) used in this study were prepared using *E. coli* BL21 as previously reported^22^. Briefly, the codon-optimized genes of the DBDs were constructed in the pET21b vector with a C-terminal His-tag. The constructed vectors were then transformed into the *E. coli* BL21 (DE3) strain. The transformed bacteria were induced by adding isopropyl β-D-1-thiogalactopyranoside to a final concentration of 0.5 mM and then grown overnight at 18 °C to express the DBDs. To purify the DBDs, the bacterial cells were collected and resuspended in buffer A (25 mM HEPES, pH 7.0, 1.0 M NaCl), followed by sonication and centrifugation. Afterward, DBDs in the supernatant were purified using a Ni-chelating column and size-exclusive chromatography (Superdex 75, GE Healthcare, USA), and the DBDs were finally eluted in buffer C (25 mM HEPES, pH 7.0, 100 mM NaCl). The purified DBDs were stored at − 80 °C after flash freezing in liquid nitrogen.

### KaScape procedures

**Figure 1:**
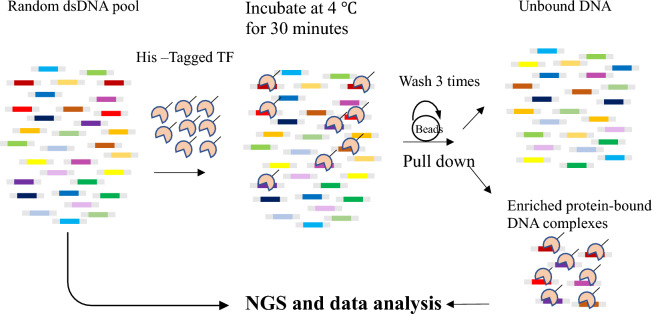
Schematic description of the KaScape process, not to scale. The random dsDNA pool (n = 4,5,6, or 7), represented by colored rectangular bars above, and the His-tagged TF DBD were prepared. The pooled dsDNAs consisted of approximately 30 base pairs with flanking sequences (Table S1). Next, $2\times {10}^{-11}$ mol protein and ${10}^{-10}$ mol dsDNA were mixed in 2 mL buffer. The TF-DBD and dsDNAs were then incubated for 30 min. Magnetic His-tag purification beads were added, and rotation was performed for one hour, and the system was then washed and rotated 3 times. The dsDNA and TF-DBD complexes were then separated from the free unbound dsDNAs. Finally, the random dsDNA pool and bound dsDNAs were extended and used to produce the dsDNA library separately for next-generation sequencing.

The KaScape procedure consists of the following five steps (Fig. 1):

#### Mixing protein with dsDNA

For each of the *At*WRKY proteins, $2\times {10}^{-11}$ mol DBDs and ${10}^{-10}$ mol random dsDNA pool (approximately 1 µg) were mixed with buffer C to a final volume of 2 mL in an EP tube and incubated on ice for 30 min.

#### Separation of bound and unbound dsDNA

First, the magnetic His-tag purification beads (BeaverBeads™ IDA-Nickel, Beaver for Life Science, China) were balanced with buffer C according to the product instructions. Second, 10 µL of balanced magnetic beads was added to the protein-dsDNA mixture. Third, the mixture was gently rotated (approximately 10 rpm) for one hour at 4 °C using an HS-3 vertical mixer (SCIENTZ, China). Fourth, after the magnetic beads were clearly separated from the mixture on a magnetic stand, the supernatant was slowly removed with a pipette. Fifth, the magnetic beads were suspended in 1 mL buffer C by rotating at 10 rpm for one hour using an HS-3 vertical mixer (SCIENTZ, China). Sixth, after the magnetic beads were clearly separated from the mixture on a magnetic stand, the supernatant was slowly removed with a pipette. The fifth and sixth steps were repeated a total of three times. Seventh, the magnetic beads were suspended in 50 µL of 500 mM imidazole by pipetting and incubated for 2 min at room temperature. Eighth, after the magnetic beads were clearly separated from the mixture on a magnetic stand, the supernatant containing protein‒DNA complexes was transferred to a new EP tube. The bound dsDNA was purified from the transformed supernatant using Oligo Clean & Concentrator Kits (ZYMO Research, USA) by following the kit instructions.

#### Extension of dsDNA

The components of the random dsDNA pool and purified bound dsDNAs were extended to 75 bp for the random sequences in which the random base length (n) was 4 (76–79 bp for n equal to 5–7), by PCR using an extension primer (Table S1 extension primer; synthesized by Sangon, China). The purified bound dsDNAs were mixed with the extension primer and EasyTaq PCR SuperMix (reagent concentrations are listed in Table S4). The mixture was then incubated using a thermocycler for polymerase chain reaction (PCR) with the program shown in Table S5. The extended dsDNAs were purified using DNA Clean & Concentrator Kits (ZYMO Research, USA) according to the kit instructions.

#### Library preparation and sequencing

Customized Illumina sequencing adapters (Table S6) were ligated to the extended random dsDNA pool and extended bound dsDNAs; ligation mix solutions were prepared as shown in Table S7 and incubated at 25 °C for 20 min. The ligated dsDNAs were purified using AMPure XP beads (Beckman Coulter, USA) by following the product instructions with a 1:1.5 ratio of the ligated dsDNAs to AMPure XP beads. For each ligated random dsDNA pool and the bound dsDNAs after purification, different Illumina indexes were added by PCR. PCR solutions were prepared as shown in Table S8, and the PCR program is shown in Table S9. Finally, the indexed libraries were purified twice using AMPure XP beads (Beckman Coulter, USA) by following the product instructions, with a 1:1 ratio between the library and AMPure XP beads. The purified libraries were sequenced on the Illumina NovaSeq PE150 platform.

#### Analysis of sequencing data

The random sequences between the designed fixed sequences were extracted from read 1 and read 2. If the extracted random sequences in read 1 and read 2 in the same pair were not reverse complements, the pair of reads was discarded. For the remaining reads of the sequencing results obtained from the random dsDNA pool, the number of reads for each type ${S}_{i}$ of random sequences was counted as ${R}_{{S}_{i}}$. For the remaining reads of the sequencing results obtained from the bound dsDNAs, the number of reads for each type, ${S}_{i}$, of random sequences was counted as ${B}_{{S}_{i}}$. Then, the proportion of sequence ${S}_{i}$ in the random dsDNA pool was calculated as $\mathrm{P}\left({S}_{i}\right)=\frac{{R}_{{S}_{i}}}{\sum_{i=1}^{{4}^{n}}{R}_{{S}_{i}}};$ while the proportion of sequence ${S}_{i}$ in the bound dsDNAs was calculated as $\mathrm{P}({S}_{i}|\mathrm{bound})=\frac{{B}_{{S}_{i}}}{\sum_{i=1}^{{4}^{n}}{B}_{{S}_{i}}}$. Finally, the relative binding energy which represents the affinity^8^ of sequence ${S}_{i}$ was calculated as ${\Delta \Delta G}_{{S}_{i}}=-{log}_{2}\frac{\mathrm{P}({S}_{i}|\mathrm{bound})}{\mathrm{P}({S}_{i})}$. The above data analysis was performed using custom code written in Python 3.6. The following modules were used: re, os, sys, datetime, collections, gc, pandas, numpy, math, matplotlib, pickle, and xlwt.

### The K-mer graph

The theory of K-mer graphs has been described in previous studies^23–26^. The 1-mer, 2-mer, 3-mer, and 4-mer graphs are shown in Fig. S1.

### KGViewer visualization software

The KGViewer visualization software (Fig. 3) was written in Python 3.6. The scientific visualization tool MayaVi^27^ and the Python graphical user interface display tool QtGui were used.

## Results and discussions

### Development of KaScape

To accurately determine the binding ability of all types of dsDNA to a protein under the same experimental conditions, measuring the fraction of each type of dsDNA sequence bound to a protein in the same liquid mixture is an ideal solution if the initial dsDNA distribution is uniform. To achieve this goal, a dsDNA pool containing all types of dsDNA sequences is needed, which can be generated by random ssDNA synthesis^14^. Since the binding sites of most single-domain transcription factors are short (for example, the length of TFBS bound by WRKY is approximately 6 bp^28^), we designed a random dsDNA pool with 4, 5, 6, and 7 random bases. Ideally, each bound dsDNA molecule should be bound by only one transcription factor, which should be located exactly in the random base region. The more a specific type of DNA is measured, the stronger the binding signal for that type of dsDNA will be. Based on these assumptions, we developed a new method called KaScape (Fig. 1), which can determine the relative affinity landscape of a given transcription factor. Theoretically, the affinity of a given TF-DNA interaction can be determined as the binding energy:

$$\Delta G=-RTln\left(\frac{[TF\cdot {DNA}_{bound}]}{\left[{TF}_{unbound}\right][{DNA}_{unbound}]}\right)$$

Several groups have established that molecular counting of DNA via HTS can measure bound and input concentrations and simplify the relative binding affinity as the relative binding energy^8,17^:

$$\Delta \Delta G=-RTln\frac{{P}_{bound}}{{P}_{input}}$$

We adopted this simplified relative binding energy to represent the relative binding affinity (see “Analysis of sequencing data” section).

**Figure 2:**
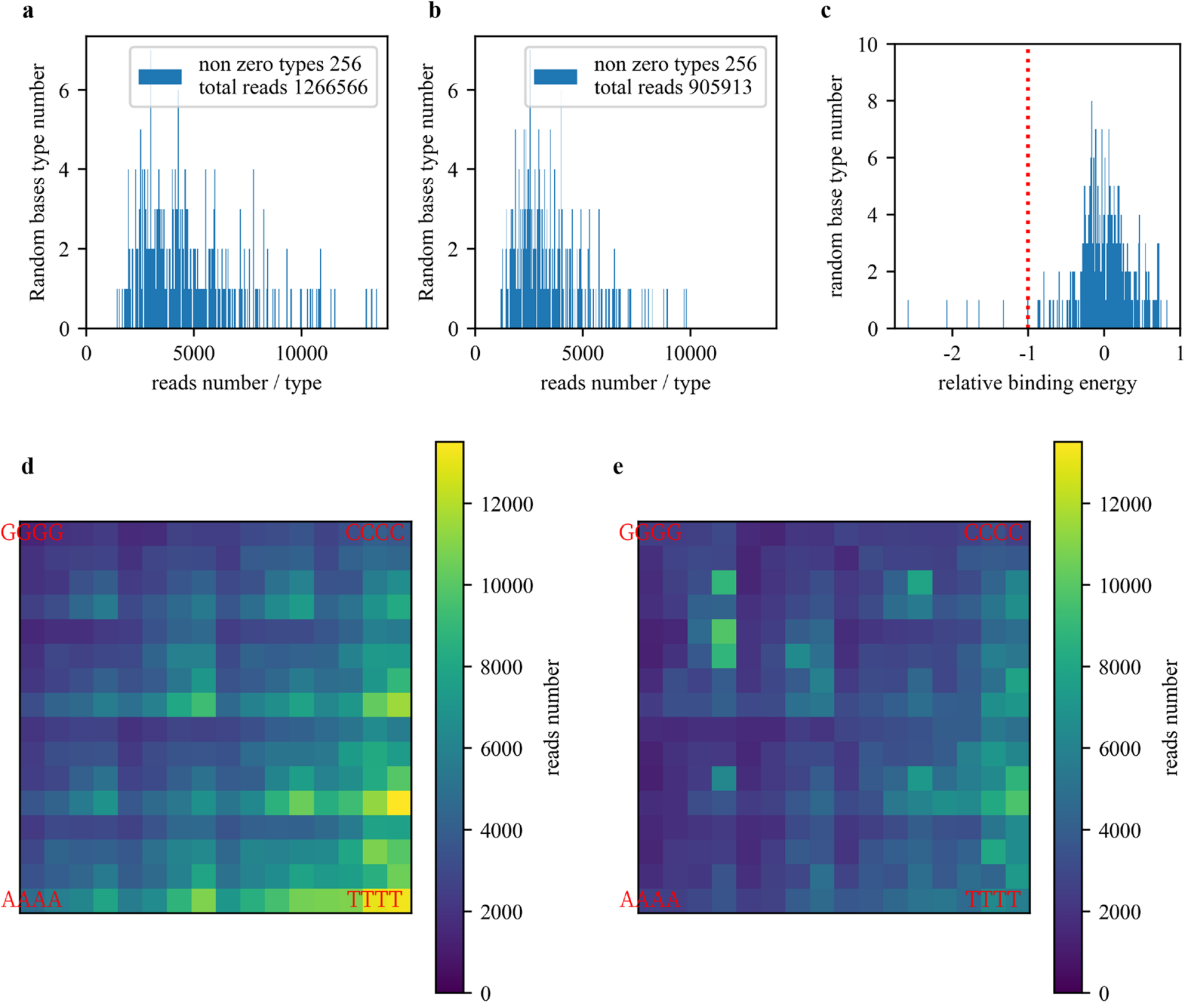
KaScape raw sequencing data. The random base number in the DNA sequence is 4. (**a**) The random dsDNA pool distribution. Each bar represents the random base type number for a given range of read counts. (**b**) The bound DNA distribution. (**c**) The relative DNA binding energy distribution. The red line is the cutoff for high affinity signals. (**d**) The read depth landscape of the random dsDNA pool in the K-mer graph. (**e**) The read depth landscape of the bound dsDNAs in the K-mer graph.

In a KaScape experiment, we first prepared a DNA pool containing all possible sequences with a given random base length n (n can be 4, 5, 6, or 7) using randomized ssDNA synthesis and dsDNA generation (see “Randomized dsDNA preparation” section). The length of the oligonucleotide used for binding experiments was approximately 30 nucleotides (nt), and the Tm of the complementary ssDNA (see Table S1) was estimated to be approximately 60 °C. To assess the uniformity of the random dsDNA pool, the pool was sequenced (see below). We calculated the distribution of the random dsDNA pool (Fig. 2a) from the sequencing results (2.3.5). Figure 2a shows a slight bias, and most differences in abundance between sequences were < 6. The read depth bias is due to the base usage in ssDNA synthesis, with the usage order T > A > C > G (Fig. 2d). Next, we incubated the randomized dsDNA pool with the transcription factor DBD (see “Protein preparation” and “Mixing protein with dsDNA” sections). Our preliminary experiments showed that a slight excess of DNA over protein is more appropriate. Too much initial DNA results in the final data being dominated by high-content sequences from the initial DNA library due to the uneven distribution of input DNA. On the other hand, if too little DNA is used, the specific DNA will not be able to stand out among the nonspecific DNA sequences due to experimental noise. The amount of protein used was determined by the amount of DNA in a 1:5 molar ratio. Since DNA may be lost in each step, the initial amount of DNA is approximately 1 µg. The longer the length of the randomized region, the more DNA is needed. One microgram is sufficient for DNA with a random base length of 4–7 nt. To remove as much nonspecific binding as possible, we washed the DNA‒protein complexes three times (see “Separation of bound and unbound dsDNA” section). We found that washing once, twice, or three times in KaScape did not significantly change the results (Fig. S2b); the washing step was the only essential step that altered the protein‒DNA binding equilibrium for different sequences in our design.

The bound DNA was then isolated by pull-down (see “Separation of bound and unbound dsDNA” section). Due to the uneven random dsDNA pool, both the random dsDNA pool and bound dsDNA libraries had to be prepared. The length of the dsDNA was too short to build a library for NGS. Therefore, before preparing the dsDNA library, we extended the random dsDNAs and the bound dsDNAs into DNA fragments of more than 70 bp each (see “Extension of dsDNA” section). There was 15 nt of overlap between the extension primer (see Table S1) and the dsDNA to ensure extension efficiency. The Taq DNA polymerase used in the extension step has terminal transferase activity, resulting in the addition of a single nucleotide (adenosine) at the 3’ end of the extension product, which is convenient for subsequent library construction. The extended dsDNA was easily ligated to sequencing adapters without the need for the “end repair” and “adenylation” steps required by many commercial NGS kits. This method results in time and cost savings compared to commercial kits. Finally, the random dsDNA pool and bound dsDNA libraries were constructed and sequenced by next-generation sequencing (see “Library preparation and sequencing” section). We then analyzed (2.3.5) the distribution of the bound dsDNAs (Fig. 2b). Figure 2b shows that the distribution was narrower and the peak was shifted to the left compared to the random dsDNA pool distribution (Fig. 2a). Several dsDNA sequence types were highly enriched in the bound dsDNAs (compare Fig. 2d and e). However, there was also a similar pattern in both the random dsDNA pool and the bound dsDNA read depth landscape due to nonspecific binding (compare Fig. 2d and e). We used the relative binding energy (2.3.5) to characterize the relative affinity. Based on the relative binding energy values (Fig. 2c), it appeared that most of the relative binding energies were greater than 0, and there was a normal distribution in the range of − 1 to 1. Notably, there were several values significantly lower than − 1, indicating that the sequences were strongly bound (Fig. 2c). We confirmed that the relative binding energy values of all sequences were highly reproducible in repeated experiments (Fig. S2a).

Since next-generation sequencing depth is critical, we evaluated sequencing depths for KaScape by simulation. We adopted the sequencing depth requirement calculation from^8^, as shown in Fig. S3. If the random base length of dsDNAs is 4, the library size is 256, and the ddG range between dsDNA and protein is assumed to be between 0.5 and 5 kcal/mol, we need more than 100,000 reads to achieve close to 100% accuracy. For the paired-end 150 sequencing strategy (PE150), where each read pair occupies 300 bases, one needs at least 30 (300 $\times$ 100,000) M for a KaScape experiment when the random base length is 4. To investigate the sequencing depth requirement based on experimental data, we calculated the correlation coefficient of the relative binding energy distribution between the experimentally derived data and several randomly downsampled simulated data (see Fig. S4). The results were similar (compare Figs. S3 and S4).

Therefore, the KaScape method robustly characterizes the relative binding landscape of all possible TFBSs simultaneously.

### KGViewer, K-mer-based 3-D visualization software

**Figure 3:**
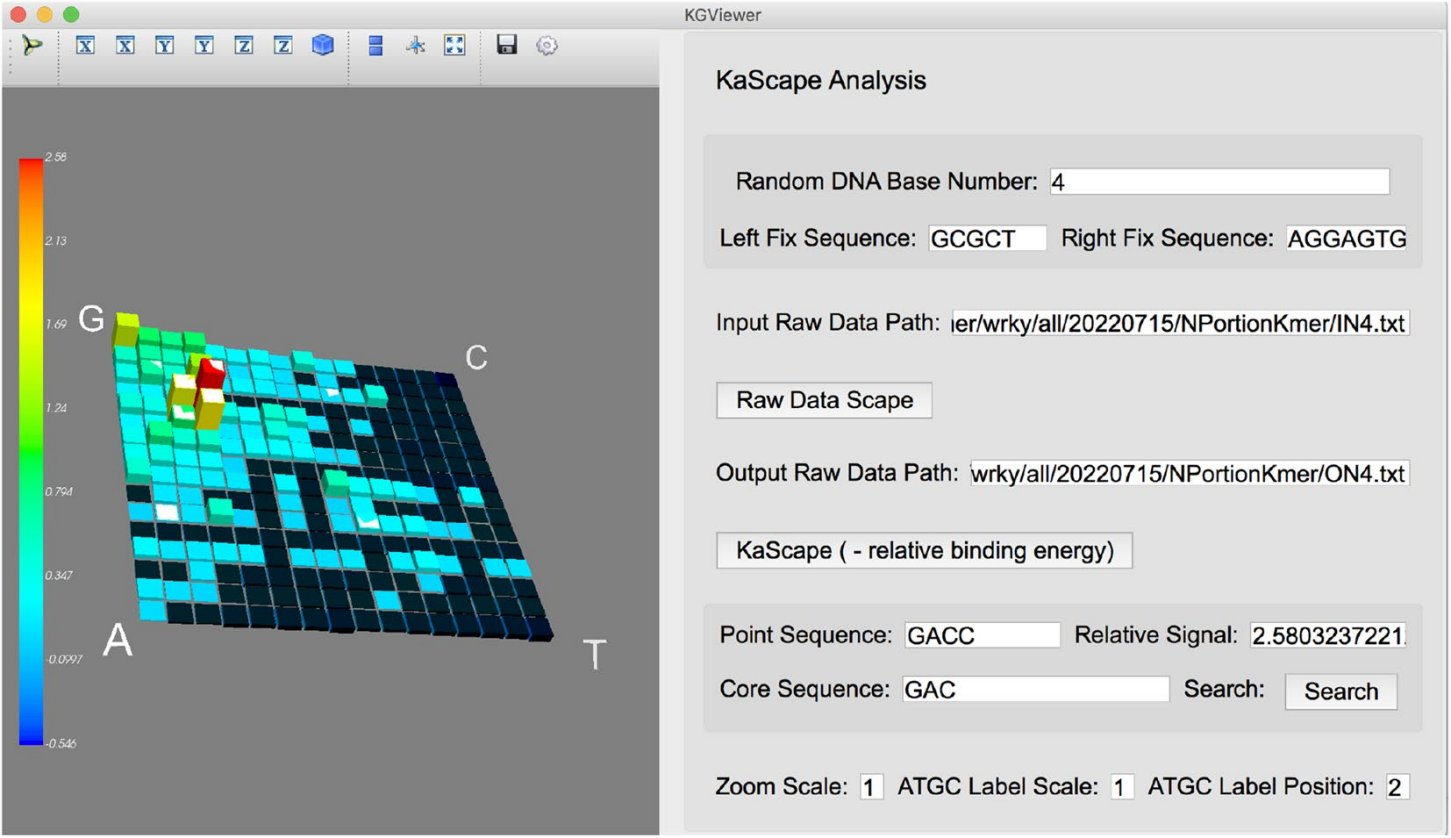
3-D KaScape viewing software KGViewer. The distribution of the random dsDNA pool and the relative binding affinity landscape can be viewed in the K-mer graph on the left. The sequence and signal values can be seen by clicking on the color bar (for example, GACC and 2.58). The highlighted sequence (see the bright bar) contains the core sequence searched by the user (here, NGAC or GACN is highlighted by searching the core sequence GAC, N represents one of the four bases).

To study the interaction of dsDNAs with a DBD protein (e.g., protein recognition and binding to a specific DNA sequence), the best in vitro approach is to measure a certain value (e.g., affinity or—relative binding energy, as used in this paper, see “Analysis of sequencing data” section) exhaustively for all possible DNA sequences under thermodynamic equilibrium conditions (3.1). However, the global comparison of a measured value for all possible sequences (when the length is > 2) is difficult. To directly compare a value among all possible sequences, we developed software called KGViewer (Kmer-Graph Viewer) (see “KGViewer visualization software” section and Fig. 3). KGViewer can visualize the given values of all possible sequences by the height and color of the bars plotted in a K-mer-based graph (Fig. S1).

The KGViewer has the following features. The size of the K-mer-based graph (2, 3, 4, 5, 6, and 7 bases) can be adjusted by setting the “Random DNA Base Number” parameter. The random dsDNA pool distribution landscape can be conveniently visualized in a K-mer graph in the left panel by specifying the input raw data path (the path to the random dsDNA pool distribution landscape file) and clicking the "Raw Data Scape" button. After specifying the output raw data path (the path to the bound dsDNA distribution landscape file) and clicking the “KaScape (- relative binding energy)” button, the left panel will change to show the minus relative binding energy landscape. To view the value and sequence information of a bar in the landscape, the user can click on the bar, which will display the relevant information in the "Point Sequence" and "Relative Signal" text boxes. For example, clicking on a bar may display information such as the sequence 'GACC' and a value of 2.58. To visualize all sequences that contain a core sequence (e.g., ‘GAC’) of interest, a search function has been provided to highlight them (e.g., ‘NGAC’ or ‘GACN’, containing GAC, is highlighted, where N represents one of four bases). The landscape size, the size of the G, C, A, and T labels and their positions can be scaled in the last row of the right panel.

This flexible KGViewer software can visualize and directly compare the values of all possible fixed-length sequences in a single plot, which is useful for studying the distribution of a value in a K-mer-based space.

### Binding affinity landscape for WRKY proteins

**Figure 4:**
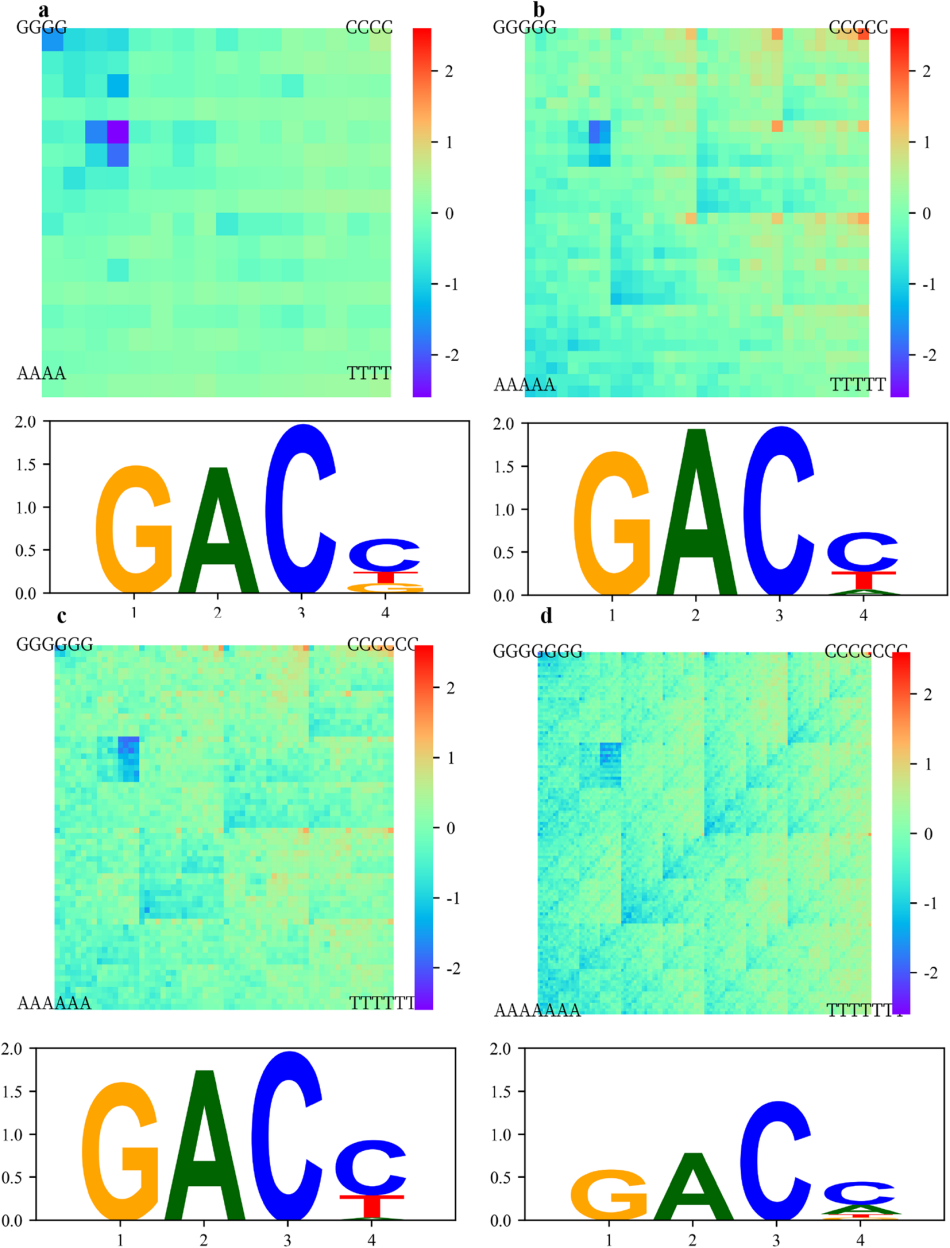
*At*WRKY1N binding specificity characterized by KaScape experiments with a series of dsDNA sequences of random base length. Upper figures are the binding energy landscape on a K-mer graph. Lower figures are the corresponding PWM sequence logo. The poorly informative positions are dropped. The sequences with relative binding energies of less than − 1 (also see the red line cutoff in Fig. 2c) are used to generate the PWM sequence logo. (**a**) The random base length of the sequences used in the KaScape experiment is 4. (**b**) The random base length of the sequences used in the KaScape experiment is 5. (**c**) The random base length of the sequences used in the KaScape experiment is 6. (**d**) The random base length of the sequences used in the KaScape experiment is 7.

We applied KaScape to the proteins of the WRKY family (Fig. S5). To evaluate the overall binding affinity of the N-terminal domain of *Arabidopsis* WRKY1 (*At*WRKY1N), we constructed a series of KaScape experiments using random base lengths (n) of 4, 5, 6, or 7. To facilitate the interpretation of the binding affinity data obtained from the KaScape experiments, a K-mer graph was used to arrange the relative binding energies in a clear and concise manner (Fig. 4). The relative binding energy shows a proportional decrease as the color shifts from red to purple. The four relative binding energy landscape maps show similar patterns. To assess the consistency across the series of KaScape experiments, we derived the K-mer relative binding energy landscape map from the (K + 1)-mer relative binding energy landscape map (Fig. S6). Comparing Fig. S6a–c with Fig. 4a–c, respectively, shows that the patterns are similar. The correlation coefficients of Fig. S6a and Fig. 4a, Fig. S6b and Fig. 4b, and Fig. S6c and Fig. 4c are 0.18, 0.88 and 0.75, respectively. The results of the correlation coefficient analysis suggest that the KaScape experiments conducted for random base lengths of 5, 6, and 7 are highly consistent. The signals in the random-base-length 4 KaScape experiments produce a cleaner relative binding energy map (compare Fig. 4a and S6a). In Fig. 4, a small region of low relative binding energies is consistently observed in the upper left portion of all K-mer graph plots. The sequences within this region correspond to GACN, GACNN, GACNNN, and GACNNNN (N represents one of the four nucleotides (G, C, A, or T)), where n is 4, 5, 6, or 7. When n is 4, the relative binding energy order in the upper left signal region is as follows: GACC < GACT < GACG < GACA (Fig. 4a). This tendency remained as n became larger. For example, when n was 5, the relative binding energy order was generally GACCN < GACTN < GACGN < GACAN.

**Figure 5:**
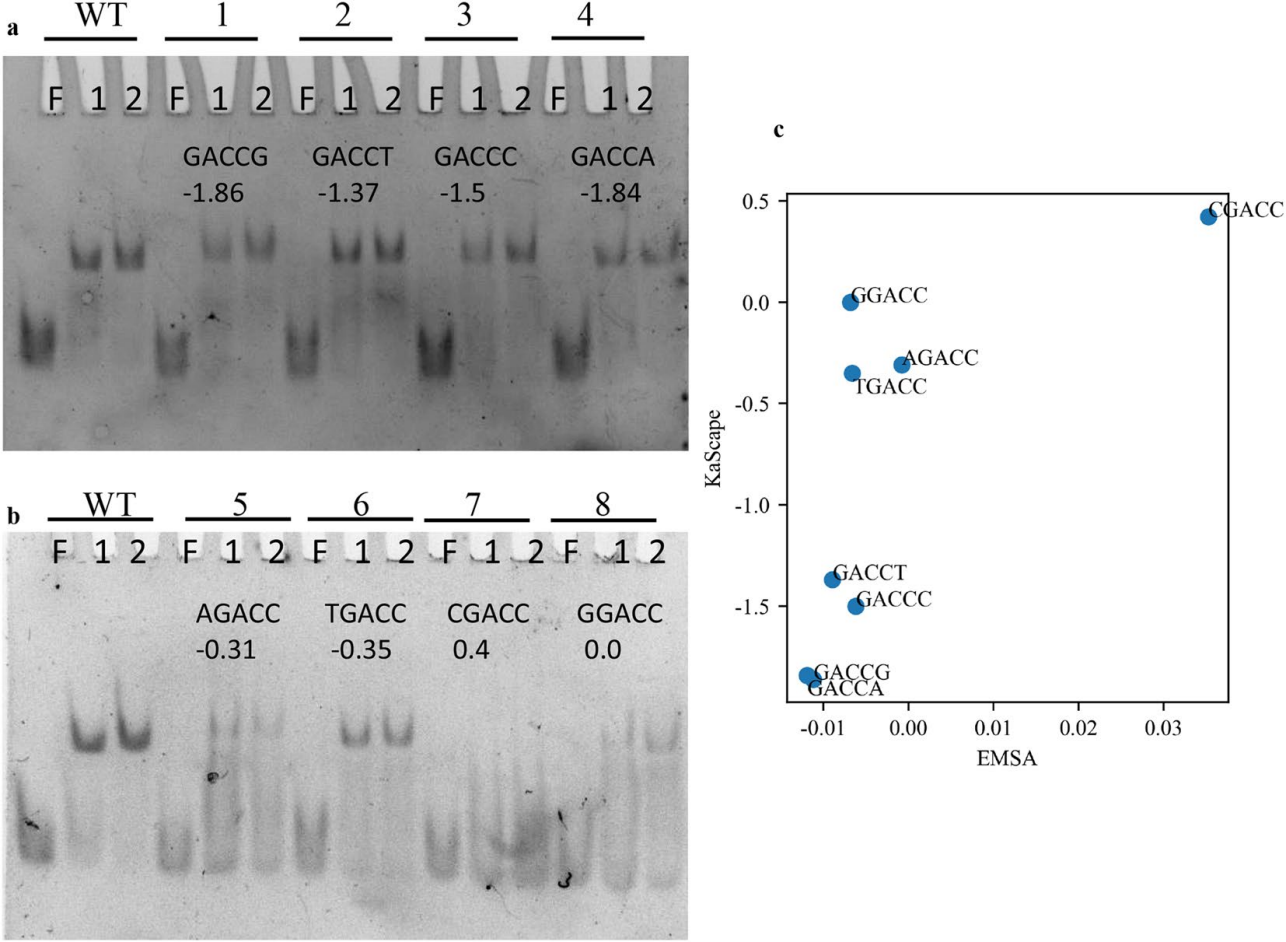
Comparison between KaScape and EMSA results for *At*WRKY1N. (**a,b**) The top row represents different dsDNA sequence types. The WT dsDNA sequence type contains the W-box sequence TTGACC. The type 1 dsDNA sequence is GCGCTGACCGAGGAG. The type 2 dsDNA sequence is GCGCTGACCTAGGAG. The type 3 dsDNA sequence is GCGCTGACCCAGGAG. The type 4 dsDNA sequence is GCGCTGACCAAGGAG. The type 5 dsDNA sequence is GCGCTAGACCAGGAG. The type 6 dsDNA sequence is GCGCTTGACCAGGAG. The type 7 dsDNA sequence is GCGCTCGACCAGGAG. The type 8 dsDNA sequence is GCGCTGGACCAGGAG. In the second row, F represents free dsDNA, and 1,2 indicates that the molar ratios of protein to DNA are 1:1 and 2:1, respectively. The third row is the sequence type. The number in the last row is the relative binding energy value calculated in the KaScape experiment. (**c**) Quantitative comparison between KaScape and EMSA results. The y-axis value represents the relative binding energy calculated from the KaScape experiment. The x-axis value is calculated as − log2(bound/unbound). The bound and unbound are the mean grayscale values in the bound and unbound region quantified from the EMSA experiment.

To confirm the KaScape results, we performed EMSA experiments for 8 sequences containing GACCN or NGACC (N represents G, C, A, or T) (Fig. 5). *At*WRKY1N is able to bind all of these sequences except the sequence containing CGACC. The relative binding energy of CGACC was 0.4, which was the highest among the 8 sequence types used in the EMSA experiment. The relative binding energy of the other 7 sequence types was less than or equal to − 0.0 (Fig. 5a, b). The EMSA results are in agreement with the KaScape results (Fig. 5c). To gain insight into the binding specificity of the sequences with the highest affinities, we used PWM sequence logos to analyze the KaScape experimental data. Figure 4 shows the sequence logos of the lowest relative binding energy sequences from each KaScape experiment of different random base lengths. The cutoff relative binding energy is − 1. The specific sequences are “GAC(C/T)”, “GAC(C/T)”, “GACC(C/T)” and “GACC” when n is 4, 5, 6, and 7, respectively. They are consistent across the series of KaScape experiments. Recognition of the W-box “TTGAC(C/T)”^28^ by the WRKY domain has been reported, but subsequent studies revealed that the predominant binding contribution came from a shorter core sequence, “GAC” (or “GTC” in reverse complement), for the WRKY family in general^22,29^ (Fig. S5). The PWM sequence logos for WRKY1 generated by PBM and SELEX are shown in Fig. S7. These results are consistent with previously reported papers^22,28,29^. The core sequence of a particular protein is defined as the bases with high information in the PWM sequence logo. The high-information sequence in the PBM sequence logo is GAC, whereas the high-information sequence in SELEX is TTGACC. The length of the core sequence in PBM data is shorter than that in SELEX data because in SELEX, only high-affinity sequences can be generated, whereas in PBM, the range of affinities generated is wider. The core sequence generated in the KaScape experiment is also GAC, which is consistent with PBM. The KaScape experiment is easier to perform. The core sequence information is already included in the random-base-length 4 KaScape experiment. In addition to the bound sequences containing the core sequence GAC, there are noncanonical motifs to which WRKY family proteins can bind, such as ‘CAACA’, which can be specifically bound by *Tamarix hispida* WRKY4 (*Th*WRKY4)^30^. The relative binding energy of ‘CAACA’ in the random-base-length 5 KaScape experiment is − 0.63. The value is less than 0, which means that ‘CAACA’ can enrich the binding of the transcription factor compared to the nonspecific binding sequences. Thus, the KaScape results can not only provide information about the core sequence of the canonical W-box motif but also provide noncanonical information^31^. By providing the relative binding energy landscape for all possible sequences, KaScape can identify not only high-affinity binding sequences but also low-affinity binding sequences^32^ that are specific to the transcription factors of interest.

**Figure 6:**
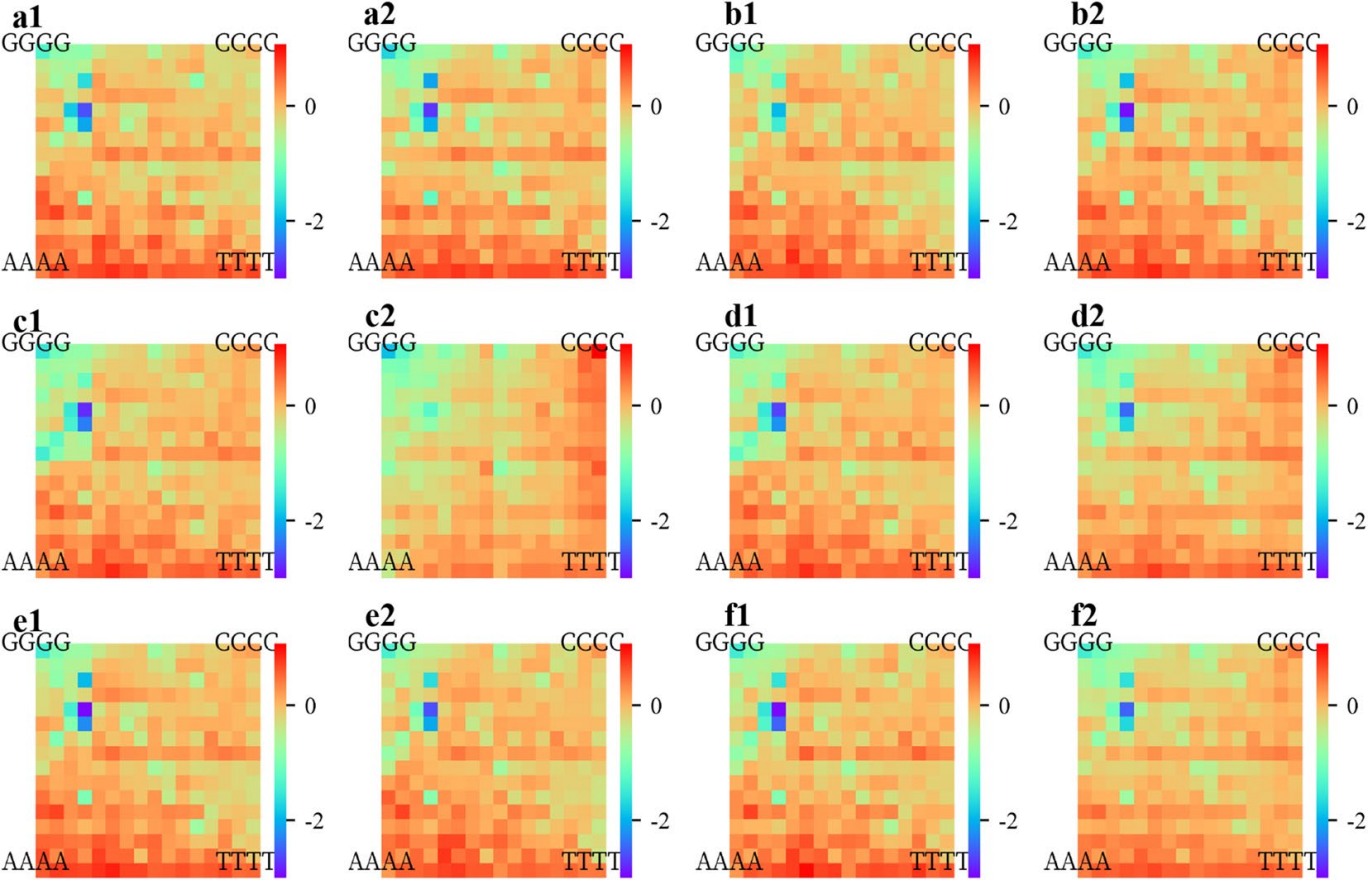
KaScape relative binding energy landscape maps for *At*WRKY family proteins. (**a1**) *At*WRKY1N, (**a2**) *At*WRKY1C, (**b1**) *At*WRKY2N, (**b2**) *At*WRKY2C, (**c1**) *At*WRKY3N, (**c2**) *At*WRKY3C, (**d1**) *At*WRKY4N, (**d2**) *At*WRKY4C, (**e1**) *At*WRKY32N, (**e2**) *At*WRKY32C, (**f1**) *At*WRKY33N, (**f2**) *At*WRKY33C. Wherein ‘N’ represents the N-terminal domain of WRKY, while ‘C’ represents the C-terminal domain of WRKY, the random base length in all subfigures is 4.

To determine whether the core sequence is conserved in WRKY family proteins, we performed 12 KaScape experiments for the N- and C-terminal DNA-binding domains of *At*WRKY1, *At*WRKY2, *At*WRKY3, *At*WRKY4, *At*WRKY32, and *At*WRKY33 (see the multiple sequence alignments in Fig. S5). Since the core sequence information is already included in the random-base-length 4 KaScape experiment (Fig. 4), the random base length of random dsDNA for the KaScape experiments used here is 4. Figure 6 shows the relative binding energy landscape maps for the *At*WRKY family proteins. The maps are similar. Most of the correlation coefficients of the relative binding energy between each pair of *At*WRKY family proteins were greater than 0.9 (Fig. S8b). This indicates that the core sequence is conserved for *At*WRKY family proteins. The correlation coefficients of the relative binding energy between *At*WRKY3C and the other 11 *At*WRKY family proteins were less than 0.8, as shown in Fig S8. In addition, the relative binding energy landscape map of *At*WRKY3C was higher than those of other *At*WRKY family proteins, as shown in Fig. 5. These observations suggest that *At*WRKY3C may exhibit weaker binding specificity and binding affinity than the other *At*WRKY family proteins tested.

## Conclusions

We developed a new NGS-based experimental method called KaScape and KGViewer software to extensively, directly, and intuitively characterize and compare the thermodynamic relative binding affinities in one landscape map. From the KaScape method, we can obtain the high- and low-affinity binding sequences for the protein of interest without the requirement for the base-independent assumption. The core sequence can also be obtained from the KaScape experiment. To explore the binding preference influence from the flanking sequence around the core sequence, the random base length can be extended to longer than 7, or the random sequence can be designed with random bases flanking the core bases. Although we only showed binding preference lengths less than or equal to 7, by including the size of the library and sequencing depth, KaScape experiments should be able to determine binding preferences for lengths greater than 7, which needs to be explored in the future. The only hurdle we can foresee for longer randomized dsDNAs, say 10–15 nt is mostly the sequencing cost which has been keeping on dropping significantly.

The KaScape method works very well for *At*WRKY family proteins, which are monomeric when binding to dsDNAs, and whether it will be equally applicable to other monomeric or even dimeric DNA-binding proteins remains to be investigated, and we see no reason why not. Last, but not least, compared to other high-throughput methods, the KaScape has more advantages such as simplicity, is easy to conduct routinely by any biological lab, and the capability of detecting a wide range of thermodynamic binding affinities and core sequences. With the current rapid development of NGS platforms and reduction of sequencing costs, the KaScape should be able to gain rapid attention and widespread application.

### Supplementary Information

Supplementary Information.

## Data Availability

The KGViewer software and the sequencing data analysis scripts are distributed freely and are available through the GitHub repository (https://github.com/NinYuan/KaScape.git).
