# Supplementary material for: KaScape: a sequencing-based method for global characterization of protein‒DNA binding affinity
Source: Sci Rep. 2023 Oct 3;13:16595. doi: 10.1038/s41598-023-43426-x (PMC10547764; doi:10.1038/s41598-023-43426-x)
Supplement: Supplementary file 1 — Supplementary Information. [file 41598_2023_43426_MOESM1_ESM.pdf]

SUPPORTING INFORMATION

KaScape: A sequencing-based method for global characterization of protein-DNA binding affinity

Hong Chen[1,*], Yongping Xu[1,*], Jianshi Jin[2], Xiao-dong Su[1]

[1]State Key Laboratory of Protein and Plant Gene Research, School of Life Sciences, and Biomedical Pioneering Innovation Center (BIOPIC), Peking University, Beijing, 100871, China.

[2]Current address: State Key Laboratory of Integrated Management of Pest Insects and Rodents, Institute of Zoology, Chinese Academy of Sciences, 1 Beichen West Road, Chaoyang District, Beijing, 100101, P. R. China

S1. SUPPLEMENTARY FIGURES

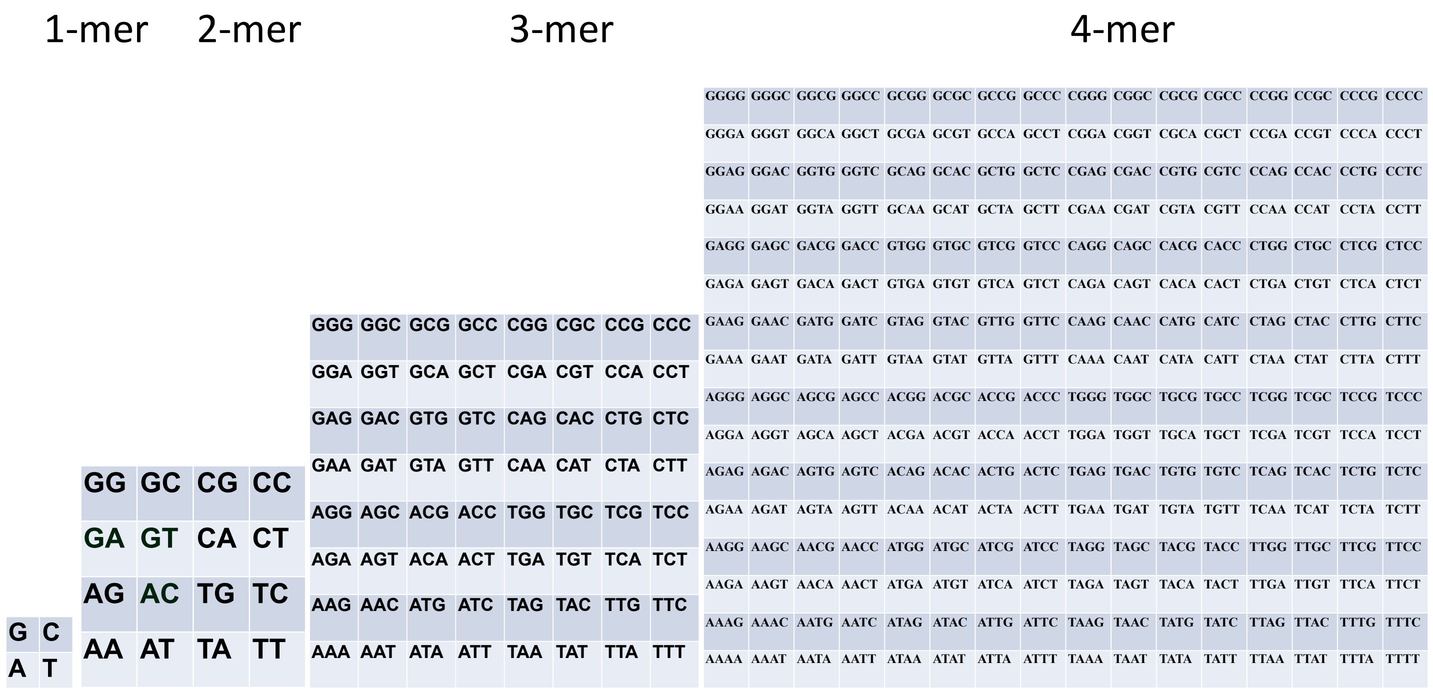

Fig. S1. K-mer graph. 1-mer, 2-mer, 3-mer, and 4-mer graphs are shown.

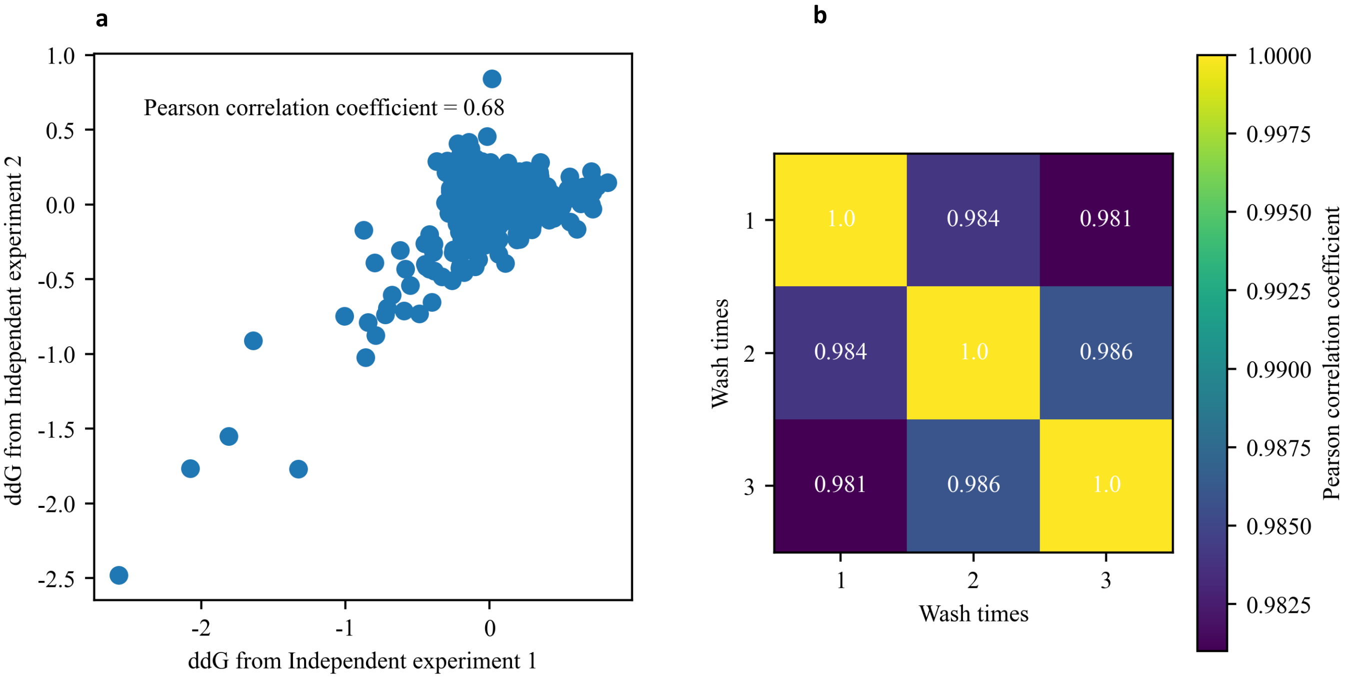

Fig. S2. Reproducibility of KaScape experiments. (**a**) Comparison of relative binding energies in two independent experiments. Pearson correlation coefficient is 0.68. (**b**) Pearson correlation coefficients of relative binding energy values in KaScape experiments with different wash times.

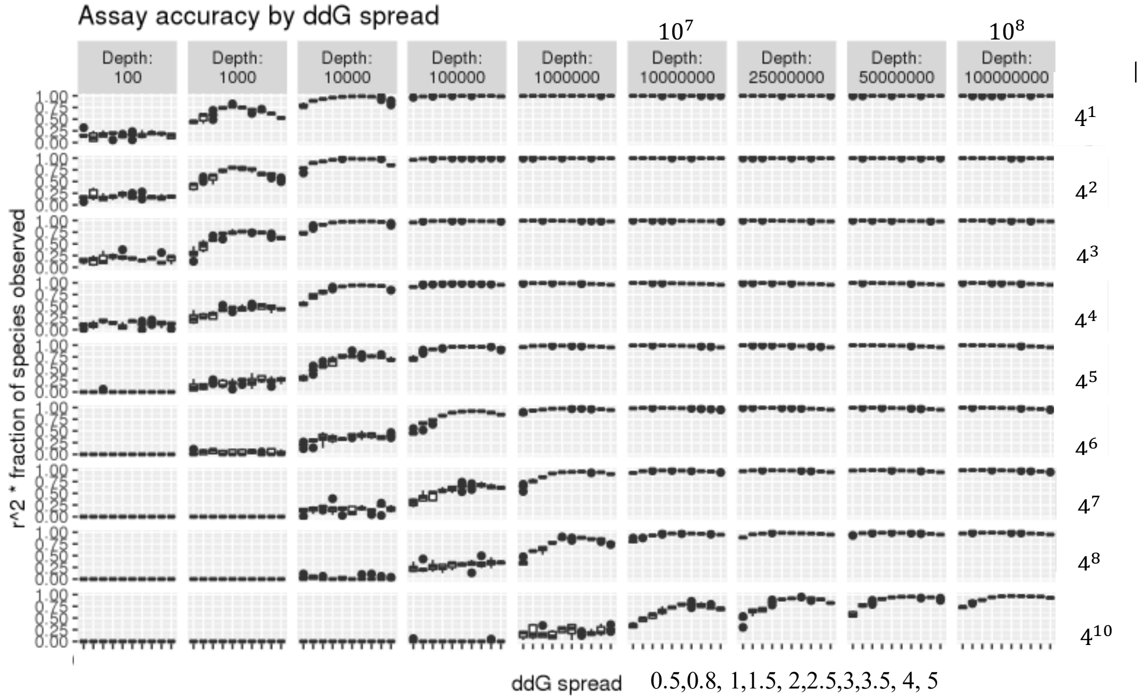

Fig. S3. Sequencing depth for a range of ddG, libSize, and accuracy. If the random base length is 4, which means the libSize is 4^4, and the ddG range for the interaction of dsDNA and protein is between 0.5 and 5 kcal/mol, to get 100% accuracy, the sequence number is at least 100000. Figure adapted from the paper [1].

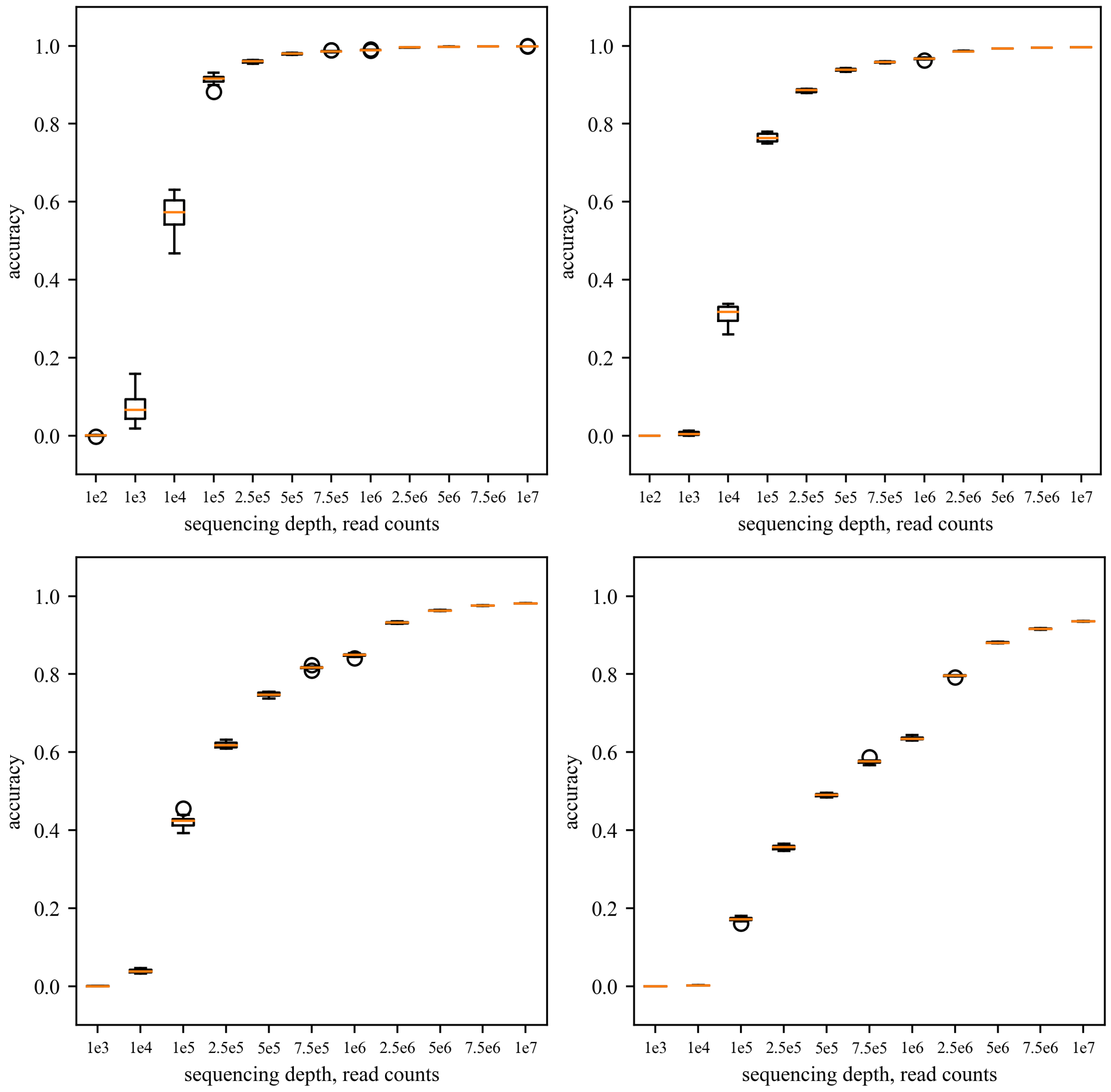

Fig. S4. Sequencing depth requirements for KaScape experiments with different random base lengths. The correlation coefficient of the relative binding energy distribution between the experimentally derived data and the randomly down-sampled simulated data. The down-sampled data are simulated 10 times for each sequencing depth. (**a**) The random base length of the sequences used in the KaScape experiments is 4. (**b**) The random base length of the sequences used in the KaScape experiments is 5. (**c**) The random base length of the sequences used in the KaScape experiments is 6. (**d**) The random base length of the sequences used in the KaScape experiments is 7.

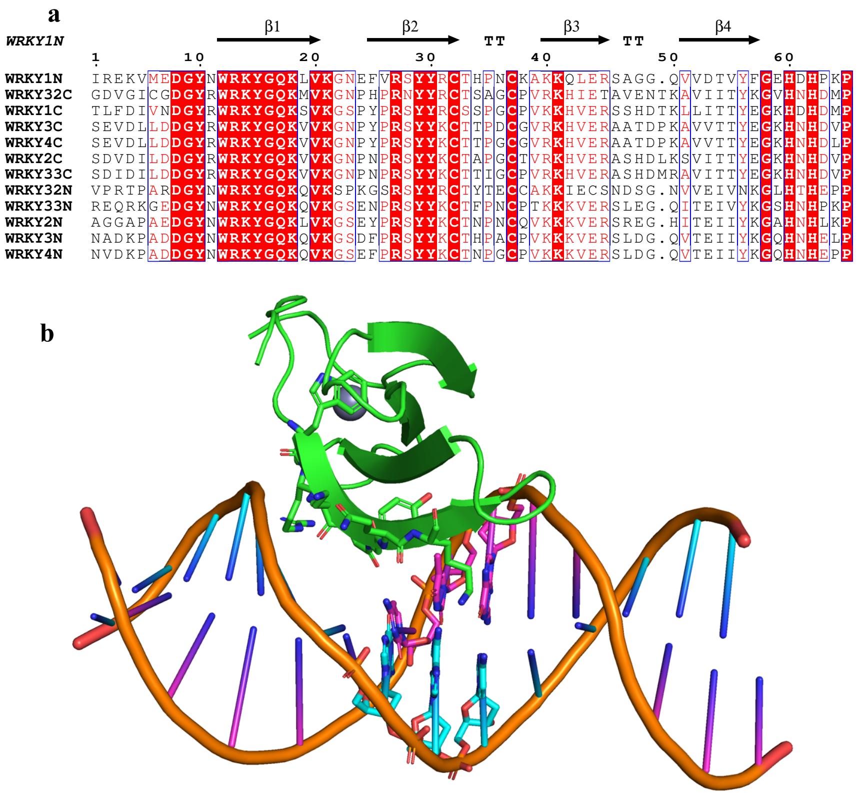

Fig. S5. A general description of WRKY. (**a**) Multiple sequence alignment of WRKY family TFs including WRKY1N, WRKY1C, WRKY2N, WRKY2C, WRKY3N, WRKY3C, WRKY4N, WRKY4C, WRKY32N, WRKY32C, WRKY33N, and WRKY33C ('N', and 'C' represent the N-terminal and C-terminal domains of the WRKY DNA-binding domain respectively). (**b**) The complex structure of the N-terminal domain of WRKY1 and dsDNA (PDB: 6J4E). The conserved motif "WRKYGQK" and the core sequence "GAC" are shown as sticks.

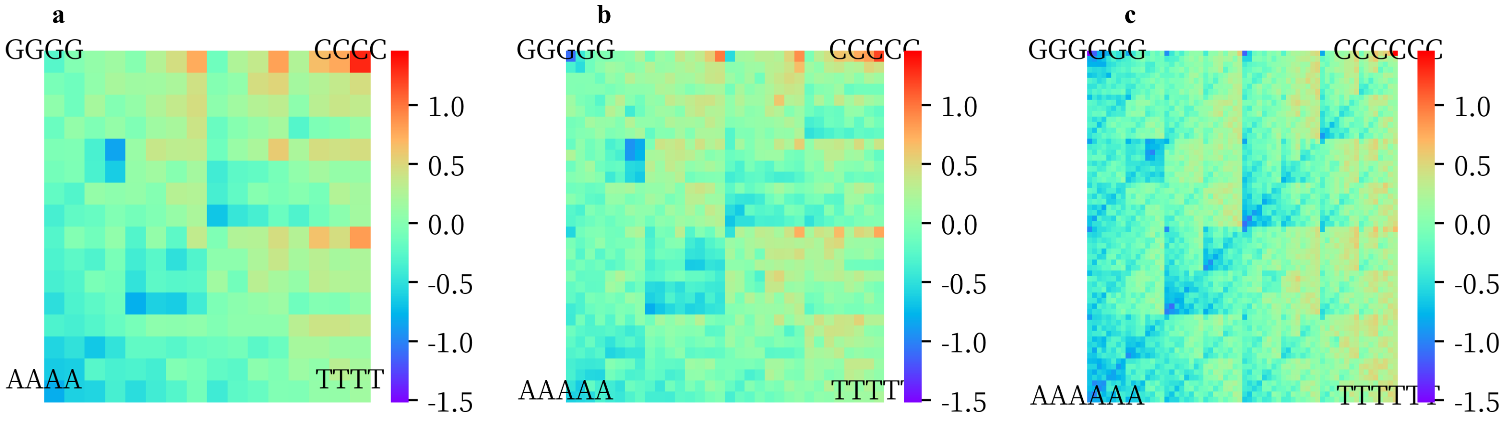

Fig. S6. The K-mer relative binding energy landscape map derived from the (K+1)-mer relative binding energy map. For a K-mer sequence, there are 8 sequence types in (K+1)-mer that can be derived to the K-mer sequence, the relative binding energy of the K-mer map is the average relative binding energy value of the 8 types. For example, for sequence GACC, there are NGACC and GACCN from 5-mer relative binding energy landscape map. The relative binding energy of GACC is the mean value of the 8 corresponding relative binding energy values in the 5-mer map. N represents G, C, A, and T. (**a**) The 4-mer relative binding energy map is generated from the random base length 5 KaScape experiment. (**b**) The 5-mer relative binding energy map is generated from the random base length 6 KaScape experiment. (**c**) The 6-mer relative binding energy map is generated from the random base length 7 KaScape experiment.

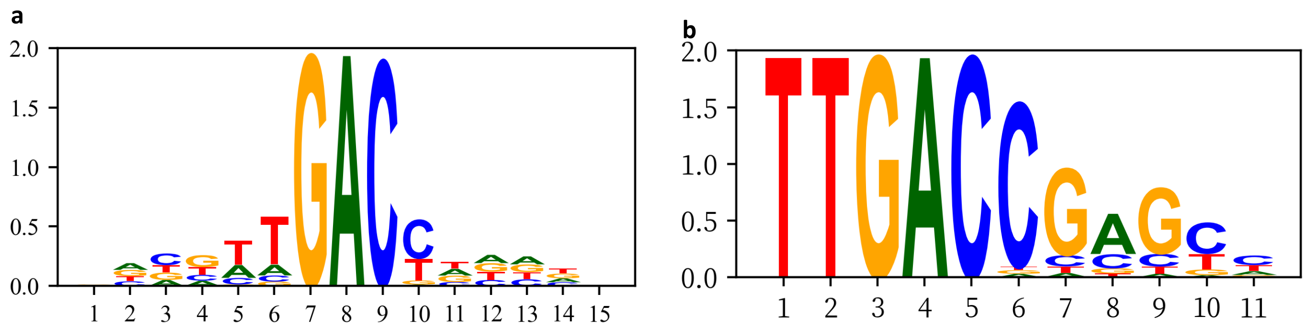

Fig. S7. PWM sequence logo from other experiments for WRKY1. (**a**) The PWM sequence logo is generated from PBM data. The 1000 highest median intensity sequences are used to generate the sequence logo. The data is downloaded from UniProbe (UP00582). (**b**) The PWM sequence logo is from the jaspar website (MA0589.1) generated by SELEX.

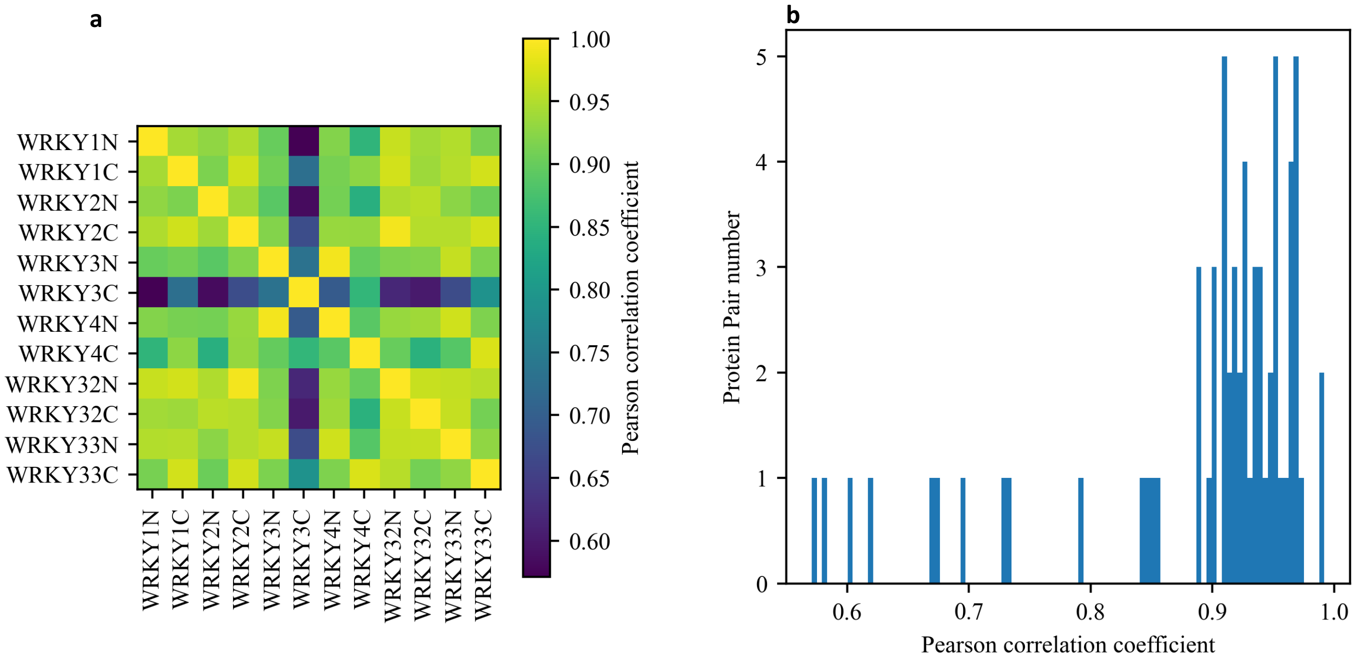

Fig. S8. The Pearson correlation coefficients of relative binding energy for *At*WRKY family proteins. (**a**) The heat map of correlation coefficients for each pair of *At*WRKY family proteins. (**b**) The histogram of Pearson correlation coefficients of relative binding energy for each pair of *At*WRKY family proteins.

S2. SUPPLEMENTARY TABLES.

Table S1 ssDNA sequence used in the KaScape

Name	DNA sequence
Random ssDNA	GCGCT$(N)_n$AGGAGTGGGATCCGGGGGGG
Complementary ssDNA	CCCCCCCCGGATCCCACTCC
Extension ssDNA	GGCGCATCAACGTGGCACTTGAGCATGCCTAAC GTAGCCGTAAGCCCCCCCCGGATCCC

Table S2 DNA Complementary system 100 µL

Material	Volume (concentration)
Random ssDNA	10 µL(100 µM)
Complementary primer	12 µL(100 µM)
2×EasyTaq PCR SuperMix	50 µL
Deionized sterilized water	28 µL

Table S3 DNA complementary program

Temperature (℃)	time	
94	5 min	
52	2 min	10 cycle
72	15 sec	
72	10min	

Table S4 DNA extension system 50 μL

Material	Volume (concentration)
dsDNA	22.5 µL
Extension primer	2.5 µL (100 µM)
2×EasyTaq PCR SuperMix	25 µL

Table S5 DNA extension program

Temperature (℃)	Time	
94	5 min	
52	2 min	5
72	15 sec	cycle
72	10 min	

Table S6 Customized adapter sequences

Name	DNA sequence
Adaptor 1F	ACACTCTTTCCCTACACGACGCTCTTCCGATCT
Adaptor 1R	GATCGGAAGAGCACACGTCT
Adaptor 2F	ACACTCTTTCCCTACACGACGCTCTTCCGATCTAT
Adaptor 2R	TAGATCGGAAGAGCACACGTCT
Adaptor 3F	ACACTCTTTCCCTACACGACGCTCTTCCGATCTCGT
Adaptor 3R	CGAGATCGGAAGAGCACACGTCT
Adaptor 4F	ACACTCTTTCCCTACACGACGCTCTTCCGATCTGAAT
Adaptor 4R	TTCAGA TCGGAAGAGCACACGTCT
Adaptor 5F	ACACTCTTTCCCTACACGACGCTCTTCCGATCTTCCAT
Adaptor 5R	TGGAAGATCGGAAGAGCACACGTCT
Adaptor 6F	ACACTCTTTCCCTACACGACGCTCTTCCGATCTATAGATT
Adaptor 6R	ATCTATAGATCGGAAGAGCACACGTCT

Table S7 Adaptor ligation system 50 *μ*L

Material	Volume
Adaptor 15 **μM**	3 μL
Extended dsDNA	37 μL
Blunt/TA Ligase Master Mix	10 μL

Table S8 Illumina index addition system 50 μL

Material	Volume (concentration)
Ligated dsDNA	22 μL
Index primer	1.5 μL (15 μM)
Universal primer	1.5 μL (15 μM)
2× KAPA SuperMix	25 μL

Table S9 PCR program

Temperature (℃)	time	
98	45 sec	
98	15 sec	6-8 cycle
60	30 sec	
72	30 sec	
72	1 min	

Table S20 primer sequence in index addition system

Name	DNA sequence
index primer	CAAGCAGAAGACGGCATACGAGATCGTGATGTGACTGGAGTTCAGACGTGTGCTCTTCCGATCT
Universal primer	AATGATACGGCGACCACCGAGATCTACACTCTTTCCCTACACGACGCTCTTCCGATCT

Reference:

1 Le, D. D. *et al.* Comprehensive, high-resolution binding energy landscapes reveal context dependencies of transcription factor binding. *Proc Natl Acad Sci U S A.* **115**, E3702-E3711, doi:10.1073/pnas.1715888115 (2018).
